# What Is a Complex Innovation System?

**DOI:** 10.1371/journal.pone.0156150

**Published:** 2016-06-03

**Authors:** J. Sylvan Katz

**Affiliations:** 1 SPRU, University of Sussex, Brighton, United Kingdom; 2 Johnson-Shoyama Graduate School of Public Policy, University of Saskatchewan, Saskatoon, SK, Canada; 3 Science Metrix, Montreal, QC, Canada; Université de Montréal, CANADA

## Abstract

Innovation systems are sometimes referred to as complex systems, something that is intuitively understood but poorly defined. A complex system dynamically evolves in non-linear ways giving it unique properties that distinguish it from other systems. In particular, a common signature of complex systems is scale-invariant emergent properties. A scale-invariant property can be identified because it is solely described by a power law function, f(x) = kx^α^, where the exponent, α, is a measure of scale-invariance. The focus of this paper is to describe and illustrate that innovation systems have properties of a complex adaptive system. In particular scale-invariant emergent properties indicative of their complex nature that can be quantified and used to inform public policy. The global research system is an example of an innovation system. Peer-reviewed publications containing knowledge are a characteristic output. Citations or references to these articles are an indirect measure of the impact the knowledge has on the research community. Peer-reviewed papers indexed in Scopus and in the Web of Science were used as data sources to produce measures of sizes and impact. These measures are used to illustrate how scale-invariant properties can be identified and quantified. It is demonstrated that the distribution of impact has a reasonable likelihood of being scale-invariant with scaling exponents that tended toward a value of less than 3.0 with the passage of time and decreasing group sizes. Scale-invariant correlations are shown between the evolution of impact and size with time and between field impact and sizes at points in time. The recursive or self-similar nature of scale-invariance suggests that any smaller innovation system within the global research system is likely to be complex with scale-invariant properties too.

## 1. Introduction

An innovation system is a network of organisations within an economic system involved in the creation, diffusion and use of scientific and technological knowledge as well as the organisations responsible for the coordination and support of these processes [1]. The concept of innovation refers to the development, adaptation, imitation and adoption of knowledge and technologies that are new to a given context. Innovation systems can found at many levels of the economy such as global, regional, national, local and sectoral levels. National systems of innovation have been a particular area of strong interest [2–4].

The design of effective innovation policy to benefit society and the economy is partially predicated on the notion that decision makers have reliable evidence-based measures to inform their decisions [5,6]. This is an area of intense investigation as witnessed by recent articles on Science Metrics in Nature and the National Science Foundation’s (NSF) The Science of Science & Innovation Policy (SciSIP) program [7,8].

Why should we care if an innovation system is or is not complex? Complex systems have unique properties that are distinctly different from those of other systems. Most, if not all, real-world complex systems possess a particular property—*scale-invariance*—that can be measured and quantified. A scale-invariant property is one that is statistically similar at many levels of observation. The structure of, and the processes taking place inside such scale-invariant systems are the same over a broad range of spatial and temporal scales[9]. Conventional measures often used as indicators of performance of an innovation system are incapable of quantifying this property [10]. If an innovation system is not complex then there is no need to worry and the current indicators will suffice. However, if an innovation system is complex it will be shown that *scale-independent* measures are needed to fully inform innovation policy. This paper focuses on the identification and quantification of scale-invariant emergent properties of an innovation system to illustrate how this unique property can be used to inform public policy.

Some people might ask if we can answer the question “Is a given innovation or research system complex?” The only answer is yes. The logic is simple [11]. An innovation system is a social construct created by a biological system of humans. Biological systems, particularly humans, are intuitively accepted as being complex adaptive systems. Innovation or research is an emergent property illustrative of their adaptive capabilities. However, this simple answer doesn’t provide any guidance as to how to identify and use naturally occurring scale-invariant characteristics of a complex innovation system to inform public policy.

The focus of this article is the general question “What is a complex innovation system?” The paper describes and illustrates how complex systems theory and techniques can be used to identify and quantify scale-invariant emergent properties. It shows how these measures can be used to inform innovation policy. First an overview of complex systems theory and techniques for identifying and quantifying scale-invariance is provided. Scale-invariant properties of innovation systems that have already been identified are reviewed. And finally the principles discussed in the preceding sections are applied to size and impact measures to illustrate how scale-invariant properties can be identified and measured.

## 2. Complex Systems and Scale-Invariance

Over the past few decades an extensive literature has been published on the study of complex physical, biological and social systems. Complex systems differ from complicated systems. Generally speaking a complicated system is understood through structural decomposition while a complex system is understood through a functional analysis [12]. Complicated systems tend to be distinctive and specialized occurring relatively rarely while complex systems tend to be generic and pervasive.

The notion of complexity and complex systems in the sciences was introduced by Weaver in 1948 [13]. He made a distinction between disorganized and organized complex systems. Disorganized complex systems are associated with extremely large physical systems like a system of gas molecules or stars in the universe. They are uniquely characterised using probability theory and statistical mechanics to create a compact description usually based on the mathematics of averages. An organized complex system simultaneously involves a large number of interrelated variables that form what Weaver called an ‘organic whole’. He claimed there are a wide variety of such systems particularly in the biological, medical, psychological and economic sciences. Moreover, he claimed they cannot be uniquely described in a compact manner by the mathematics of averages.

Complex systems have some generally accepted properties [14–16]. Their structure spans several scales. Their constituents are interdependent and interact in nonlinear ways. These interactions give rise to novel and emergent dynamics. The combination of structure and emergence is viewed as *self-organization* [17]. Self-organization which is closely related to scale invariance may in a natural way be among the most important mechanisms leading to scale invariance in complex systems [9]. Biological evolution is a form of self-organization produced by dissipative dynamic processes that decreased system entropy through the utilization of energy and molecular materials leading to increased organization and complexity [18]. The Sante Fe Institute has prepared a Complexity Explorer web site with a number of excellent on-line MOOCs that discuss complex systems and their unique properties [19].

There are a number of types of complexity such as algorithmic, computational, mathematical, physical and symbolic complexity. We will focus on physical and symbolic complexity exemplified by biological systems and human languages. These are two types of systems that most people intuitively agree are complex [11].

Humans generate speech and written words from a need to communicate meaning in a given world or social context; their utterances obey a complex system of syntactic, lexical, and semantic regularity [20]. Symbolic complexity in human language is partially exemplified by two scale-invariant properties: the Zipf distribution of words in documents, irrespective of language, and the Lotka distribution of scientific productivity, irrespective of language and culture. Lotka distributions are used to model scale-invariant properties of human information production processes such as the published output of a research system [21].

Physical complexity often involves the interplay between chaos and non-chaos producing critical points where self-organization is most likely to occur [22,23]. A complex systems is neither too ‘ordered’ nor too ‘disordered’ but finely balanced between the two [11]. In order for a complex biological system to survive and evolve there must be interplay between competition and co-operation at different scales. Furthermore, the non-linear dynamics of a complex system must be co-operative for self-organization to occur [24]. This is a fundamental characteristic of insect & animal colonies and human activities. Complex biological/social systems are called *adaptive* systems because they can adapt to a changing environment. A small subset of adaptive complex systems are *self-reproducing* and experience birth, growth and death.

*Emergent* properties are the most often observed real world phenomena in a complex system. Emergent properties are patterns and regularities arising through interactions among smaller or simpler entities in a system that themselves do not exhibit such properties. In biological systems interactions at lower levels emerge as objects expressing their properties at a higher level [25]. Emergent properties tend to arise as new objects from one scale to another. For example, life is an emergent property; none of the component molecules of a cell are alive, only a whole cell lives.

Emergent properties are a key generic property of complex adaptive economic system; it is what makes economies become complex [26]. Emergent properties appear at many levels of observation in complex systems. Consider a Romanesque broccoli—a complex biological system—that produces an edible flower constructed of elegant spiral swirls. The flower is composed of smaller florets that mimic the shape of the main flower. Each of the smaller florets is composed of even smaller florets with similar spiral swirls. This process repeats itself until near the cellular level. The spiral swirls are natural fractals that emerge during the growth of the flower. They are described by a series of fractal arcs that follow the well-known recursive Fibonacci series [27]. These fractal arcs are not found at the level of individual cells and molecules such as proteins, DNA, minerals, etc. It is an emergent property of the collective dynamic activity of these entities.

We will see that innovation systems exhibit emergent properties that can be found at many levels of observation too. For example, van Raan clustered highly cited scientific papers using co-citation relationships [28]. He showed that the size-rank distribution of these clusters had the unique signature of a fractal and the signature could be observed at different levels of aggregation. He concluded the fractal emergent properties of co-citation clusters provided a representation of the ecosystem of scientists.

An emergent property is identified by the scaling behavior of variables describing a structural feature or a dynamical characteristic of the system [15]. Scaling behaviour occurs when an identical or statistically similar property occurs at many levels of observation. This property is referred to as a *scale-invariant* property because it appears to be statistically similar irrespective of scale. It is commonly associated with things like the *self-similar* structure of geometrical and natural fractals [29].

There are other common terms synonymous for scale-invariance most notably *cumulative advantage*, *Matthew Effect*, *Yule process* and *preferential attachment* [30]. They have their roots in the Gibrat's law or Gibrat's rule of proportionate growth or the law of proportionate effect [31]. Merton called this success-breeds-success phenomenon by which the rich get richer while the poor get comparatively poorer the “Matthew Effect,” after a well-known verse in the Gospel according Matthew [32,33]. de Solla-Price coined the term cumulative advantage to describe processes that produce scale-invariant citation probability distributions [34]. Barabási and Albert called the processes that produce scale-invariant distributions of network links preferential attachment [35]. Cumulative advantage and preferential attachment are different names for the same process; both are based on a Polya urn model [36]. It is important to note that preferential attachment in datasets does not imply that preferential attachment is the active mechanism. It simply implies that past activity is correlated with whatever growth mechanism is actually at play. Preferential attachment is an effective mechanism that reproduces the statistical properties of scale-invariant growth [37].

Scale-invariance can be perfect, as in the case of a deterministically defined geometrical fractal, or it can be statistical, as in the case of jaggedness of an island shoreline or the billowiness of clouds in the sky [29,38]. Scaling properties can be measured and used to characterise attributes of a complex system. Measures based on scale-invariant properties are called *scale-independent or scale-adjusted measures* [39]. These measures have been used to make comparisons between cities with scaling properties and provide meaningful rankings of urban systems [40]. Unlike conventional per capita indicators scale-independent measures are dimensionless and independent of size. There have been other attempts to build size-independent measures however, this paper focuses solely on scale-independent measures useful for characterizing scale-invariant properties [41,42].

**Fig 1:**
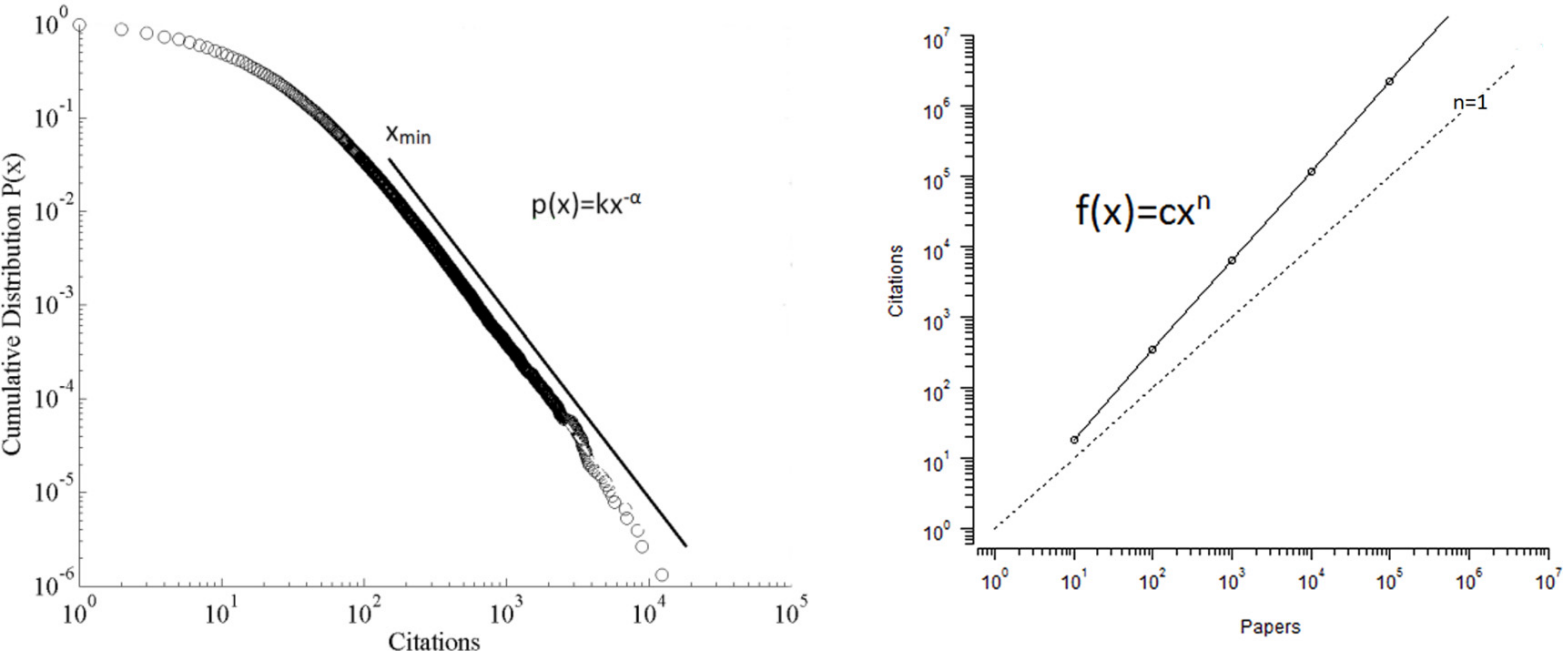
Power law (a) probability distribution and (b) correlation.

Scale-invariance is mathematically defined as p(bx) = g(b)p(x) for any b [30]. In other words, if we increase the scale or units by which we measure x by a factor of b, the shape of the distribution p(x) is unchanged, except for an overall multiplicative constant. Two mathematical functions that posses this property are shown in Fig 1: (a) power law probability distributions defined by p(x) = kx^-α^ for x≥x_min_, the value of x at which the power law tail of the distribution begins, and (b) power law correlations, defined by f(x) = cx^n^, where k & c are constants. While f(x)≡p(x) when n = -α, x_min_ = 1 & α>0 for illustrative purposes distributions and correlations are discussed separately. Power law functions are characterized by their linear appearance on a log-log scale. The exponents n and α, also called *scaling factors*, are measures of the scale-invariance of properties. **From this point forward the symbol α will be used to denote the exponent of either a scaling distribution or correlation**

A common comment about power law probability distributions is that items in the tail of the distribution only account for a small fraction, sometimes less than 1%, of the total population [43]. There are two primary reasons why research has focused on the tail. The shape of the tail determines if the distribution is or is not scale-invariant. Moreover, the tail not the body of the distribution defines its variance. Later we will see that the exponent of the tail of a power law distribution determines if it can be characterized by a mean and variance. Also, we will review attempts to fit the whole distribution with a modified power law.

In order to reliably determine if the tail of the distribution is scale-invariant the tail should cover a considerable range of probabilities, P(x), and x values beyond x_min_ [44]. Consider x_min_ values for the 1997 & 1998 Scopus and 1988 Web of Science whose details are given later. The x_min_ values for Scopus evolved from 8 and 6 citations, respectively, received in the first year of publication to 435 and 463 by the end of the observation period. The most highly cited papers in these years received 36,671 and 14,769 citations, respectively. Similar values occurred for the WoS. For example, x_min_ values for the 1984 & 1985 WoS data evolved from 4 and 5, respectively, to 313 and 267 citations with the highest cited papers receiving 14,276 and 18,154 citations by the end of the observation period. The range between x_min_ and x_max_ in the tails of the WoS and Scopus distributions are close to 3 orders of magnitude and the range of P(x) for Scopus and WoS was close to 4 orders of magnitude. This is illustrated in a dynamic graphic of the evolution of the 1984 & 1985 WoS impact distributions [45]. The length of the tails in these distributions is sufficient for making a reliable determination of their characteristics.

**Fig 2:**
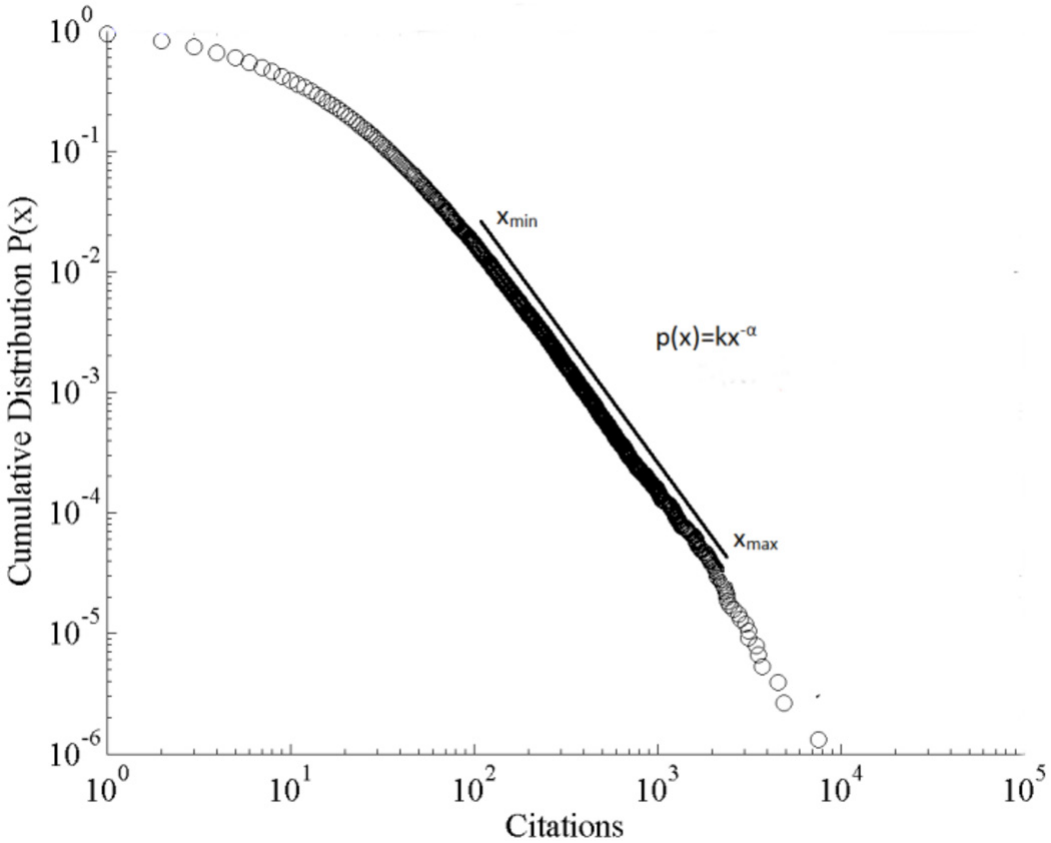
Power law with exponential cut-off.

Fig 2 shows a degenerate form of a power law distribution called a power law with exponential cut-off given by f(x) = k x^*−*α^e^*−*λx^. In these cases some entities in the far right hand tail of the distribution do not occur with as high a probability as would be expected of a pure power law distribution. For a pure power law scale-invariance is found for all x>x_min._ Scale-invariance for a power law with exponential cut-off is limited to the region x_max_>x>x_min_ where x_max_ is the point at which the exponential cut-off starts to dominate the power law. The region of scale-invariance between x_min_ and x_max_ can be several orders of magnitude in size.

Some research suggests that the exponential cut-off of the power law is due to finite size of the data set, but recently it has been shown that it might also be an effect of finite observation time [46]. And some models also show that the probability distribution tends to evolve from exponential to a power law with exponential cut-off to a pure power law given enough time.

Random and natural populations drawn from a scale-invariant distribution are scale-invariant too. A natural population is one that preserves its clustering, ‘community’ or small world structure [47,48]. Hence a scale-invariant property found in a large complex innovation system likely will occur for any natural smaller innovation system within it. It gets more difficult to statistically confirm the scale-invariance as system size decreases.

A number of physical processes are known to generate power law distributions. For example, they can be generated by combinations of exponential distributions [30,49,50]. They can be generated using the same multiplicative process that generates lognormal distributions by imposing lower bounds [51]. This is one reason it is difficult to distinguish a power law from lognormal distribution. Other power law generating mechanisms include specific Yule processes such as preferential attachment and critical phenomena that occur with continuous phase transitions [30,35]. History-dependent processes are ubiquitous in nature and social systems and frequently exhibit scale-invariant properties [52]. History-dependent stochastic process associated with complex systems become more constrained as they unfold and their state space or set of possible outcomes decreases as they age. This reduction of state space over time leads to the emergence of power laws. The universal nature of power law distributions may be partially explained by the diversity of mechanisms that can produce them.

The existence of scale-invariant properties maybe necessary but it is not sufficient to define a complex innovation system. Scale-invariance is associated with simple and complex physical systems as well as complex biological/social systems [11]. In addition to scale-invariant properties a complex biological/social system needs to be distinguishable from a physical system. A physical system only has to do one thing–“be”–it doesn’t make choices and it is constrained by physical laws to find a state of least energy or action. A biological/social system has to make choices consistent with the restrictions imposed by the laws of physics. A complex physical system evolves solely in state space while a complex biological/social system evolves in spaces of state and strategy allowing it to adapt. By definition an innovation system is a biological/social system that makes choices adapting itself and its environment to changing knowledge, practices and technologies and as well as to changing economic, social and political forces in which it is embedded.

## 3. Innovation Systems and Scaling Properties

Innovation is an interactive process between many actors, including companies, universities and research institutes [53]. Innovation does not follow a linear path that begins with research and then moves through development, design, engineering and production resulting with the introduction of new products and processes. It is a non-linear process with feedback between the different stages of development. Innovation is an emergent property of a complex adaptive social system [54–60].

The character of an innovation system emerges from the interactions between its members and the members of other systems. Some of the interactions are more “rule-like” than others because they are governed by laws, regulations, treaties, etc. Other interactions are more random because they are governed by personal, social, political and economic forces. Economic emergence in an innovation system, that is the appearance of economic structures that cannot be explained by examining their components, requires that their analysis be fully embedded in complex economic system theory [26,61].

Leydesdorff and Etzkowitz pioneered the use of the metaphor ‘Triple Helix’ to describe a complex innovation system composed of university, governmental and industrial organizations. It was the seed for many publications on the topic in the social studies of science [62–64]. Recently there have been attempts to model the self-organizing character of the Triple Helix to show how fractal structures of an innovation system are replicated by innovation activities at various scales [65].

Many scaling properties have been observed for innovation systems. For example, the distribution of stock price fluctuations for US companies [66]. The growth dynamics of business and university research activities are scale-invariant [67,68]. A recent study of the European aerospace research area shows a variety of scale-invariant topologies in joint venture networks [69]. Also, a variety of scale-independent measures has been used to explore the Chinese innovation system [70]. These researchers found that some important developments were hidden by rankings using conventional performance measures that were revealed using scale-independent rankings.

Cities are society’s predominant engine of innovation and wealth creation [71]. Scaling correlations occur between city sizes and such thing as new patents issued, numbers of inventors, GDP, number of R&D establishments, private sector R&D employment and overall R&D employment [72,73]. Generally speaking, the properties of most socioeconomic systems are strongly predicted by scaling laws that are non-linear functions of population size [40].

Scale-invariant models composed of a small number of power law correlations have been constructed of the evolution of the European, Canada and Chinese innovation systems. The models were constructed using the scaling correlations between GERD & GDP and GDP & population [39,74]. They will be discussed in detail later.

Patent based indicators have been used and accepted as measures of innovation. A scaling correlation has been shown between firm R&D expenditures and number of patents issued [75]. Recently, the distribution of patents among applicants within OECD countries was shown to be scale-invariant [76]. A variety of scale-invariant properties have been found for patent measures [77].

Some measures used to investigate innovation systems are self-reported, statistically sampled and/or incomplete. Sometimes they are reported in different units requiring conversion before they can used for comparative purposes. Such data tend to be noisy and inaccurate making them difficult to analyse. The quality of these data makes distributional analysis particularly difficult especially when the data are disaggregated into smaller groups.

On the other hand, some properties such as impact and size measured using numbers of citations to peer-reviewed publications and numbers of papers published by a group are accurate and relatively noise free. Some well tested schemes are available to agglomerate publications into things like research fields and subfields facilitating the investigations of the effects of scale. Large datasets covering decades of published research are available making them ideal for illustrating scale-invariant emergent properties of an evolutionary innovation system.

Peer-reviewed publications often containing new knowledge are a common output of the global research system based in institutions and organizations like universities, hospitals, government research labs, private sector labs, etc. References or citations to these articles are used as an imperfect but quantifiable indirect measure of the impact this knowledge has on the community. Peer-reviewed publications and citations to them are examples of clean, reliable and reproducible measures of size and impact that can be used to explore scale-invariant properties of the global research system. The same principles can be applied to other measures discussed earlier.

An interesting property of scale-invariance is its recursive or self-similar character allowing properties seen at one level observation to be seen a lower levels too. For example, if we know that a property of the global research system is scale-invariant then any regional, national, local, sectoral, institutional (e.g. universities) [78], etc research system within it will have that scale-invariant property too. Moreover, if the global research system is a complex innovation system then smaller research systems contained within it are complex too.

### 3.1 Scaling Distributions

Citation distributions have been the object of intense investigation for fifty years. Price reported the right skewed nature of these distributions and later proposed a ‘cumulative advantage’ mechanism to explain the power law nature of these heavy tailed distributions [34,79]. However, until recently a robust methodology for determining the likelihood of a real-world distribution having a power law tail had not been generally accepted.

In the past a standard method for determining if a distribution was a power law was to use linear regression methods on log transformed data. However, Rousseau and others had suggested that the maximum likelihood estimation technique was better [80]. In 2005 an in-depth article reviewed the theories and empirical evidence for the existence of power-laws as well as generating mechanisms proposed to explain them [30]. In 2009 an excellent paper appeared in SIAM that detailed a comprehensive methodology for determining the existence of a power law distribution which is gaining wide acceptance [44,81]. The article examined the theory, practice and difficulties for determining the best fit model to real-world empirical data suspected of having power law probability distributions. It convincingly showed that the use of a linear regression of log transformed data is flawed and that the maximum likelihood estimation technique could be used to draw meaningful conclusions. Also the authors supplied the R:, C and Matlab routines required for the analysis.

A few papers have appeared that used the Clauset et.al techniques to explore citation distributions. Two papers looked at field level citation distributions for 1998–2002 WoS and Scopus data [43,82]. Albarrán and colleagues used WoS data and Brzezinski used Scopus data. Both groups used a 5 year fixed citation window to create the citation distributions and both encountered a similar difficulty. The WoS and Scopus field/subfield assignments allow a journal, hence an article, to be assigned to more than one field/subfield. This has the potential to change the shape of the tail of the citation distributions and warrants further investigation.

Albarrán et.al. reported that the distribution for 17 of 22 (77%) fields and 140 of 219 (64%) subfields had reasonable likelihood of being power law distribitions. The authors did not check to see if any other heavy tailed distributions might fit the data. Brzezinski reported the citation distributions of 14 of 27 (52%) Scopus fields had a reasonable likelihood of being power law distributions. He computed the log-likelihood ratio as describe in Clauset et.al to see if a given distribution was best fit by a power law or another heavy tailed distributions.

Using the same methodology a longitudinal study examined the evolution of the citation distributions to peer-reviewed papers indexed in the WoS between 1984 and 2002 and cited from the year of publication to 2009 [10]. This approach provided observation windows ranging from 6 to 25 years. Articles were uniquely assigned to one of 13 fields using the National Science Foundation (NSF) journal classification scheme so that citation distributions at the field level could be explored. For the evolution of the overall citation distribution it was found that those scaling exponents, α, with significant p-values (i.e. p ≥ 0.10) declined with time. As the distribution evolved it approached a value near 3.0, a value first reported by Redner [83]. Also, the citation distributions for 7 of 13 (54%) fields and 124 of 276 (45%) subfields had scaling exponents < 3.0 with significant p-values. In addition, 71 of the 139 Scopus subject areas had exponents < 3.0 one or more times during the first five years of the evolution of their citation distributions. This time frame is frequently used to prepare comparative impact measures.

Other studies have reported few subfields with exponents <3.0, for example, the work of Brzezinski and Albarrán et al. discussed earlier. However, these studies combined multiple years of publications and their citation counts. Citation growth and decay rates for individual years can differ and it is unclear what effect mixing citation distributions for multiple years has on the exponent of a scale-invariant tail. It is possible that it contributed to the larger exponents reported by these authors. In this study only citation distributions to individual years of publications were examined to avoid this problem.

The magnitude of the exponent of the tail of a power law distribution is important. It tells us when the mean and the variance of the distribution can be used to characterize it and when it cannot. Power law distributions with exponents > 3.0 can be characterize by their mean and variance. However, the second moment of real-world power law distributions with 2<α≤3 is infinite hence the variance is infinite [30]. Unlike power law distributions with α>3.0 they don’t reside in the domain of attraction of Gaussian distributions; hence, the Central Limit Theorem no longer applies and population averages cannot be used to characterise them [84]. The population average is only significant when the variance is finite.

Real data are finite and have a finite sample maximum but given any growing population with a maximum x value at a point in time there is a non-negligible chance at some later time the maximum value will be exceeded. If one calculates the means of random samples drawn from a power law distribution with α<3.0 the values of the means will have a power law distribution and vary over many orders of magnitude [30].

Population averages cannot accurately characterize real-world distributions with α<3.0. Many traditional measures used for comparative and evaluative purposes are valid only for Gaussian population distributions and power law distributions with α≥3.0 [85]. Such measures are poor indicators of the emerging properties of real-world complex systems [39].

Over the past few years attempts have been made to find models that fit the whole distribution not just the tail. For example, it is generally accepted that a power law distribution modeled using preferential attachment in a growing network realistically characterizes the in-degree distribution of Web links. However, some low connectivity regions such as university home web pages seem to be better characterized by a modified power law of the form p(x) = A/(B+x)^α^ where A is chosen to ensure $\sum_{x\;=\;x_{min}}^{\infty }p\left(x\right)\;=\;1,\;$ and B, the mode, and α, the exponent, are constants [86]. The location of the mode is proportional to the rate at which links are added and it appears in regions of low connectivity. When B = 0 the modified power law is a pure power law. The modified power law is modeled using a mixture of preferential attachment where links are created because of popularity and uniform attachment were links are created because of personal interests independent of popularity. The body seems to be best modeled by a log-normal distribution and the tail by a power law with a transition in between determined by a mixture of attachment types.

A simplified form of the modified power law called a *hooked power law* given by p(x) = 1/(B+x)^α^ where B>-1 was used to explore citations distributions in smaller size Scopus subject areas [87]. When x>B the tail of the hooked power law follows a power law. The authors used data consisting of papers indexed in 2004 from 20 small Scopus subject areas that accumulated citations over a 10 year period. 9 of the 20 datasets were truncated at 5000 papers due to limitations of the Scopus interface. A comparison of fits of a power law, log-normal and a hooked power to the tails of impact distributions was done for x>x_min_ where x_min_ was determined using Clauset et al. methodology. All functional forms seemed to fit equally well; however, it can be difficult to distinguish power law forms from log-normal fits particularly for small data sets [44]. When the fits were compared over the whole distribution with x_min_ = 1 the hooked power law distribution was a better overall fit. This is not a surprising finding as Clauset et al. warns if that if x_min_ is too small or too large it produces a biased estimate of the scaling parameter and a poor fit will result.

The authors also reported 8 of the 20 subfield has exponents <3.0 when the Clauset et al. methodology was used to fit the tail of the distributions for x>x_min_. On the other hand when the hooked power was used to fit the whole distribution for x_min_ = 1 the overall fit was good but only 2 of the 20 had exponents <3.0. The authors did not provided a mathematical reason or statistical evidence for the differences. They suggested that the difference is related to the hook in the power law but they did not provide any evidence that its tail fitted the range of values for x>x_min_ better or even as well as the Clauset et al. technique did. It is possible that while the hooked power fits the larger data set it is a poor fit to the tail. This is a critical factor as the exponent of the power law tail determines the variance of the distribution.

In contrast to the above approaches others have shown that papers in the body of citation distributions are dominated by direct citations while the tail is dominated by indirect citations with a ‘tipping point’ between them [88]. A direct citation is a reference to the source article and an indirect citation is a reference to an article that contains a reference to the source article in its bibliography. Papers having few citations are frequently directly cited whereas papers having many citations (e.g. “classics”) are indirectly cited. Cumulative advantage arises because there are more routes through the reference lists of intermediate papers for finding a classic paper than for finding a non-classic paper. Irrespective of which model is used to fit the whole distribution—preferential & uniform attachment, hooked power law or direct & indirect citing—they all have a scale-invariant tails.

Many innovation networks have small world properties where the diameter of the network d ≈ log N and N is the number of nodes in the network [89]. The diameter of a network is the average distance between nodes in the network. Complex networks like citation networks tend to have scale-invariant degree distributions. When the magnitude of the exponent is in the range 2<α≤3 the average diameter of the network shrinks from logN to loglogN. So if N = 10^10^ the mean distance between nodes shrinks from 10 when α>3 to 1 when α<3. The network becomes an ultra-small world network [90].

Until recently it was difficult to examine the evolution of large citation distributions. Usually a snapshot is taken of the distribution at a point in time. These data are analyzed and conclusions drawn about the likelihood that the distribution is power law distribution or another heavy-tailed distribution (e.g. log-normal, Poisson, stretched exponential). It is difficult to do longitudinal studies even using modern high performance computing facilities available on most university campuses. It can take more than 24 hours to run the simulation routine used to determine the p-value for the exponent of a single power law distribution.

An innovation system is a dynamic system and its attributes change with time. Perhaps a snapshot of a distribution at a point in time is not giving us a full picture. Later, using a longitudinal approach to look at evolutionary trends, it will be shown that impact distributions may not be scale-invariant all of the time but they have a reasonable likelihood of being scale-invariant much of the time.

### 3.2 Scaling Correlations

The global research system exhibits many scaling correlations defined by f(x) = cx^α^, where c is constant. A scaling correlation with an exponent α≠1 is indicative of scale-invariant properties. When α>1.0 the correlation is superlinear indicative of a Matthew effect or cumulative advantage [32,78,91]. When α<1.0 the correlation is sublinear indicative of an inverse Matthew effect or cumulative disadvantage. When α = 1.0 linear effects are at play more indicative of random organization rather than self-organization.

Scale-invariant correlations are found across groups within an innovation system [92]. For example, using 1981–1996 ISI (now WoS) data a scaling correlation with α = 1.27±0.03 was found between the impact and sizes of subject areas. The magnitude of the scaling exponent shows that on average a doubling in size produced a 2^1.27^ or 2.4 times increase in impact. Also, scaling correlations were found between impact and sizes across 13 NSF fields and 138 NSF subfields using 1984–2002 WoS data [10]. Impact was measured by counting citations to papers using a fixed 6-year citation window. The data were summed over the time interval. The field and subfield level scaling exponents were nearly equal with α = 1.28±0.09 and α = 1.27±0.03, respectively.

The scaling factor, α, for the correlation between citations and papers is a scale-independent measure of the average *citedness* of peer-reviewed papers produced in fields & subfields of different sizes in the global research system. The term *citedness* is a fuzzy term describing a scale-invariant property akin to terms like the jaggedness of islands and billowiness of clouds measured by their fractal dimension which is the exponent of a power law relationship.

Scaling correlations with α>1 have been found between the denominators and numerators of ratios within collections of conventional measures of performance such as citations/paper, GDP/capita and GERD/GDP. Invariably, the numerator is a measure of size and it is used as a normalizing parameter. If these indicators were truly normalized for size we would expect that the scaling correlation between the denominators and numerators would be linear (i.e. α = 1) indicating effects of size have been removed. Usually they have α>1 indicating the denominators increased non-linearly with size.

The mean-normalized citations score (MNCS) and the Hirsch or h-index are two fairly recent measures used to evaluate aspects of an innovation system. They are considered to be normalized for size. van Raan showed that the field-normalized number of citations, C_n_, for 500 of the world’s largest universities scaled with their sizes, P [78]. Given that C_n_ = P*MNCS and C_n_≈ P^1.17^ then C_n_/P = MNCS ≈ P^0.17^. For a doubling in the size of a university C_n_ would be expected to increase 2.25 times and MNCS 1.13 times. In other words, while C_n_ exhibits a Matthew effect and MNCS shows an inverse Matthew effect. The h-index, h, has been used to compare the impact of such things as individuals, journals, countries and patents. A group has an index h if h published papers have at least h citations each and the other papers receive no more than h citations. Hirsh defined the h-index as h = (C/a)^1/2^ where C is total citations and a is a constant between 3 and 5 [93]. Earlier we showed that C = kP^α^, therefore substituting for C we get h = a’ P^α/2^ where a’ = (k/a)^1/2^ is constant. If α = 1.28 as in the previous example then for a doubling of size the h-index would be expected to increase 1.55 times, an inverse Matthew effect. Many conventional indicators used as comparative measures of the performance of innovation systems are biased by size [39,74].

Recently scaling correlations were used to illustrate that research collaboration increases the impact of peer-reviewed papers beyond those of single-authored papers [94,95]. The scaling correlation between impact and numbers of collaborative papers in 173 Management journals was 1.89±0.08 whereas for single-author papers it was 1.35±0.08. Moreover, a scaling correlation was found between impact and numbers of multi-author papers in 33 subfields of the natural sciences. The scaling correlation for multi-authored papers was 1.20±0.07 and for single-authored papers it was 0.85 ± 0.11. In both instances the Matthew effect was much stronger for collaborative papers resulting in greater impact than single-authored papers. In fact, in the natural sciences single-authored papers had a scaling exponent < 1.0 indicative of a cumulative disadvantage.

A variety of other scaling correlations are associated with the global research system. Numbers of in-links and sizes of University web sites and numbers of collaborative papers and total numbers of papers published by countries show scale-invariant characteristics [91,96]. Research impact has been shown to scale with size from the level of research groups and to the level of European Universities [97,98]. The sizes of universities have been shown to scale like city sizes and the published scientific output from urban centers scales with the population of the centers [78,99].

A scale-invariant correlation can be used to construct dimensionless, relative measures properly adjusted for size useful for comparative purposes [39]. For example, the expected impact, C_e_, of a group of a given size, P, in a collection of groups in a research system having a scaling factor, α, is given by C_e_≈P ^α^. The relative impact of any group in the collection is given by the ratio of its observed impact, C_o_, to its expected impact C_e_. The magnitudes of the relative impacts of groups of vastly different sizes can be compared with the confidence that effects of size have been removed.

One of the most common scale-invariant relationship occurs between any two measures that exhibit exponential growth [100]. Assume we are given two exponential growth processes x = am^pt^ and y = bm^qt^ where m is the base, t is time and a & b are constants. Let y = sx^α^ where s is a constant, then bm^qt^ = s(am^pt^)^α^ or b/s(a)^α^ = m^(pα –q)t^. Because m^(pα –q)t^ is a time-dependent variable and it cannot be equal to b/s(a)^α^, a constant, unless pα –q = 0, therefore, α = q/p and s = b/a^q/p^. This relationship holds even if the two processes are delayed in time with respect to each other or if they have different starting values at t = 0. An example of this relationship will be presented later.

The scaling exponent of such correlations gives us a measure of the relative exponential growth of the two properties. If α = 1 both are growing at the same rate and if α≠1 then one is growing faster or slower than the other. This measure can be used to inform policy. Consider the following two examples discussed below. The first example is based on the relative growth of impact and size and the second example is based on the relative growth of Gross Expenditures on R&D (GERD) and GDP.

The scaling exponents for relative exponential growths of citations and papers were determined for each of 13 NSF fields derived from 1984–2002 WoS data. The exponents ranged from α = 1.13 in physics to α = 2.92 in biology with an average of α = 1.74 [10]. Field sizes ranged from 40 thousand to 3.2 million peer-reviewed documents. The fields were ranked by the magnitude of the exponents and compared to the ranks determined using average number of citations per paper over the time interval. It was clear that the conventional measure of average number of citations per paper was not a predictor of the relative growths of impact vs size.

European and Canadian Gross Expenditures on R&D (GERD) and GDP grew exponential from 1981 to 2000. The scaling exponents for the correlation between these parameters was found to be 1.03 and 1.42 respectively [39]. Between 1995 and 2005 the Chinese innovation system had a scaling exponent of 1.67 for the same parameters [74]. These scaling exponents tell us that the relative growth of GERD for the EU was essentially linear with respect to GDP while the Canadian and Chinese GERD tended to grow 2.67 (2^1.42^) and 3.18 (2^1.67^) times for a doubling of GDP. Let us examine these findings in more detail using scale-independent models constructed from scaling correlations.

Scaling correlations were used to build scale-invariant models of the 1980–2002 European and Canadian innovation systems and a model of the 1995–2002 Chinese innovation system. Scaling correlation for the relative growth of GERD and GDP for countries, provinces and municipalities in Europe, Canada and China, respectively, were combined with the scaling correlations that occurred between GERD and GDP across these groups at points in time in these systems [39,74,101]. A small number of scale-invariant functions uniquely described the evolution of R&D expenditure and GDP of groups within these innovation systems.

Consider the model of the European innovation system. It clearly showed that shortly after the enactment of the Single European Act in 1986 the scaling correlation between GERD and GDP across EU countries dropped from approximately 1.25 to nearly 1.0. In other words, at the beginning of the period R&D investments increased nearly 2.5 times for each doubling in country size and by the end of the period R&D investment it was increasing linearly with size. R&D investment became more equitable across EU countries with integration into a single market. During the same period the scaling correlation across Canadian provinces moved up a bit from about 1.1 to 1.14 with a brief peak at around 1.2 between 1996 and 1998. On the other hand scaling correlation across Chinese provinces and municipalities rose from about 0.86 in 1995 to around 1.25 in 2002 as R&D investment became more concentrated in the larger regions. A scale-independent model can be played forward providing an overview of how a system might evolve given the current scaling trends or how it might evolve under different policy regimes.

Let’s turn our attention to illustrations of these concepts in more detail. The next two sections describe the data and methodology used to illustrate how scale-invariant properties of impact distributions evolve with time and how the scaling correlations between impact and size can provide novel insight into the character of the global research system.

## 4. Data and Methodology

Two data sets were used to examine the evolution of citation distributions and the correlations between impact and size. One data set consisted of publications indexed in Scopus in 1997 and 1998 with annual citation counts to each paper from the year of publication to 2013. These data were awarded as part of the 2013 Elsevier Bibliometric Research Program grants. They were used to study the evolution of citation distributions over 16 and 17 year time spans and consisted of more than 800,000 peer-reviewed documents each year that were cited more than 20 million times. The second data set consisted of 10.9 million peer-reviewed source documents indexed in the WoS between 1984 and 2002 with annual citation counts for each document from the year of publication to 2009 totalling about 120 million citations [10]. These data were accessed through a Research Fellowship at Science Metrix in Montreal.

A field level analysis of the evolution of citation distributions was done using three journal schemes to classify documents: Scopus, UCSD Map of Science (MAPS) and the National Science Foundation (NSF) journal classification schemes. Earlier it was mentioned that the Scopus scheme allows a journal and the articles it contains to be assigned to one or more of 27 research fields. This raised a question. Does this affect the field level citation distributions?

This question is examined by comparing the findings using the Scopus scheme to the non-overlapping MAPs and NSF schemes. The MAPS scheme was designed for visualizing maps of science [102]. It used algebraic and clustering techniques to assign journals to one of 554 unique journal clusters which were aggregated into 14 unique science areas. Journals that covered multiple fields were not used in the analysis reducing the size of the analyzed data by 10% from its original size. The NSF scheme assigns each journal hence each article to one of 13 unique fields based on citation patterns and expert opinion. It has not been updated since 2012 so articles in newer journals are not classified reducing the analyzed data set by approximately 7% from the original size.

The methodology described by Clauset et al. and the Matlab, R: and C software routines they used were used in this analysis [44,103]. In particular, the maximum likelihood estimation method (MLE) was used to determine the scaling exponents of the cumulative probability distributions. A technique devised by Clauset and colleagues was used to estimate the lower bounds (i.e. x_min_. The p-values for exponents were determined using Monte Carlo simulations. These simulations were computationally intensive taking up to 25 hours to determine one p-value running on an 8 node, 6 processors per node, Sun Microsystems, cluster using parallel Matlab. In addition likelihood ratio tests were done comparing the best-fit power law model to other heavy tailed distributions: Poisson, log-normal, exponential, stretched exponential and power with exponential cut-off. It is important to note that in Clauset et al. the authors state that for real-world data it is extremely difficult to tell the difference between log-normal and power-law behavior unless one has very large data sets.

The distributions for each year in the evolution of the overall citation distribution were assigned one of four likelihoods of being modeled by a power law (none, moderate, good or power law with exponential cut-off) as described in the Clauset paper. The assignments are based on the amount of statistical support there is for a distribution being modeled by a power law distribution. “None” indicates the data is probably not power-law distributed; “moderate” indicates that the power law is a good fit but that there are other plausible alternatives; “good” indicates that the power law is a good fit and that none of the alternatives considered is plausible. In some cases, it is noted as “with cut-off,” meaning a power law with exponential cut-off is favored over a pure power law.

## 5. Illustrations and Discussion

The first part of this section presents an analysis of the evolution of the impact of the global research system by exploring the evolution of citation distributions to peer-reviewed papers indexed in Scopus and WoS overall and at field levels. The second section presents an analysis of the scaling correlations between the growth of the research system’s impact and size over time and the correlation between field impact and field sizes measured across fields at points in time.

### 5.1 Impact Probability Distributions

Table 1 gives the results of the tests of the fit of a power law model to the evolution of the distribution of citations to 1997 and 1998 peer-reviewed documents indexed in Scopus. One of four likelihood categories was assigned to each distribution annually. Table 2 gives similar results of the tests of the fit of a power law model to the evolution of the distribution of citations to 1984 peer-reviewed documents indexed in the WoS database. This table is an example of the analysis done for each of five years of WoS data from 1984 to 1988 that had the longest evolution times. Both tables give the magnitude of the scaling exponent, α, its p-value and the log-likelihood ratios test (LR) for alternative heavy tailed distributions and their respective p-values. Positive values of LR indicate that the power-law model is favored over the alternative [44]. The final column of the table summarizes the statistical support for the power-law fit for each year in the evolution of the distributions.

**Table 1:** Test of evolution of power law distributions for citations to peer-reviewed 1997 and 1998 documents indexed in the Scopus database. Statistically significant p-values are denoted in **bold**. For each year in the evolution of the distribution p-values for the fit to the power-law model and likelihood (LR) ratios with p-values are given for alternatives distributions.

**Scopus 1997 N = 801,501**													
**Time**			**Log-normal**		**Poisson**		**Exponential**		**Stretch Exp**		**Power law + cut-off**		**Support for power law**
**years**	**α**	**p**	**LR**	**p**	**LR**	**p**	**LR**	**p**	**LR**	**p**	**LR**	**p**	
1	3.36	**0.44**	-0.92	0.36	7.12	**0.00**	5.49	**0.00**	0.50	0.62	-1.67	**0.07**	with cut-off
2	3.10	0.00	-2.14	**0.03**	13.11	**0.00**	9.52	**0.00**	-0.46	0.65	-8.21	**0.00**	with cut-off
3	3.23	**0.83**	-0.94	0.35	8.60	**0.00**	5.75	**0.00**	0.19	0.85	-1.92	**0.05**	with cut-off
4	3.24	**0.69**	-0.68	0.50	6.91	**0.00**	5.10	**0.00**	0.63	0.53	-1.01	0.16	moderate
5	3.25	**0.52**	-0.43	0.67	5.90	**0.00**	4.68	**0.00**	0.55	0.49	-0.38	0.38	moderate
6	3.23	**0.43**	-0.44	0.66	5.46	**0.00**	4.48	**0.00**	0.98	0.33	-0.27	0.46	moderate
7	3.23	**0.54**	-0.29	0.77	4.90	**0.00**	4.17	**0.00**	1.01	0.31	-0.07	0.70	moderate
8	3.23	**0.85**	-0.21	0.83	4.86	**0.00**	4.31	**0.00**	1.26	0.21	-0.01	0.87	moderate
9	3.21	**0.86**	-0.27	0.78	4.77	**0.00**	4.31	**0.00**	1.16	0.24	-0.01	0.89	moderate
10	3.17	**0.17**	-0.47	0.64	4.59	**0.00**	4.11	**0.00**	0.81	0.42	-0.04	0.79	moderate
11	3.10	0.09	-0.81	0.42	5.75	**0.00**	5.46	**0.00**	1.06	0.29	-0.35	0.40	none
12	3.11	**0.31**	-0.54	0.59	5.08	**0.00**	4.81	**0.00**	1.08	0.28	-0.08	0.68	moderate
13	3.10	**0.76**	-0.41	0.69	4.88	**0.00**	4.66	**0.00**	1.25	0.21	-0.03	0.82	moderate
14	3.10	**0.55**	-0.26	0.80	4.43	**0.00**	4.18	**0.00**	1.32	0.19	0.00	0.98	moderate
15	3.06	**0.26**	-0.48	0.63	4.88	**0.00**	4.70	**0.00**	1.19	0.23	-0.04	0.77	moderate
16	3.06	**0.24**	-0.29	0.77	4.47	**0.00**	4.27	**0.00**	1.14	0.25	0.00	0.95	moderate
17	3.06	**0.25**	-0.24	0.81	4.38	**0.00**	4.18	**0.00**	3.38	**0.00**	0.00	0.99	moderate
**Scopus 1998 N = 816,486**													
**Time**			**Log-normal**		**Poisson**		**Exponential**		**Stretch Exp**		**Power law + cut-off**		**Support for power law**
**years**	**α**	**p**	**LR**	**p**	**LR**	**p**	**LR**	**p**	**LR**	**p**	**LR**	**p**	
1	3.21	0.04	-1.72	**0.09**	9.46	**0.00**	7.56	**0.00**	n/a	n/a	-5.18	**0.00**	with cut-off
2	3.34	**0.68**	-0.72	0.47	7.77	**0.00**	4.65	**0.00**	0.02	0.99	-1.09	0.14	moderate
3	3.30	**0.51**	-0.30	0.76	9.53	**0.00**	6.49	**0.00**	1.16	0.25	-0.49	0.32	moderate
4	3.30	**0.91**	-0.15	0.88	9.60	**0.00**	6.76	**0.00**	1.46	0.14	-0.27	0.47	moderate
5	3.26	**0.83**	-0.18	0.86	9.54	**0.00**	6.85	**0.00**	1.43	0.15	-0.29	0.45	moderate
6	3.01	0.00	-2.35	**0.02**	16.26	**0.00**	12.60	**0.00**	0.15	0.88	-8.37	**0.00**	with cut-off
7	3.01	0.00	-2.05	**0.04**	14.93	**0.00**	11.62	**0.00**	0.48	0.63	-5.95	**0.00**	with cut-off
8	3.15	**0.71**	-0.13	0.90	9.27	**0.00**	7.20	**0.00**	1.74	**0.08**	-0.27	0.47	moderate
9	3.11	**0.72**	-0.13	0.90	9.48	**0.00**	7.51	**0.00**	1.86	**0.06**	-0.29	0.44	moderate
10	3.06	**0.64**	-0.29	0.78	10.74	**0.00**	8.79	**0.00**	1.96	**0.05**	-0.47	0.33	moderate
11	3.05	**0.73**	-0.11	0.91	10.97	**0.00**	9.22	**0.00**	2.39	**0.02**	-0.31	0.43	moderate
12	3.04	**0.76**	-0.05	0.96	10.87	**0.00**	9.22	**0.00**	2.39	**0.02**	-0.27	0.47	moderate
13	3.02	**0.56**	-0.09	0.93	10.85	**0.00**	9.22	**0.00**	2.44	**0.01**	-0.30	0.44	moderate
14	3.01	**0.27**	0.14	0.88	10.63	**0.00**	9.07	**0.00**	2.53	**0.01**	-0.22	0.51	good
15	2.99	**0.69**	0.15	0.88	9.63	**0.00**	7.96	**0.00**	5.17	**0.00**	-0.30	0.44	good
16	2.99	**0.54**	0.25	0.80	9.70	**0.00**	8.06	**0.00**	5.28	**0.00**	-0.27	0.46	good

**Table 2:** Test of evolution of power law distributions for citations to peer-reviewed 1984 documents indexed in the Web of Science database. Statistically significant p-values are denoted in **bold**. For each year in the evolution of the distribution p-values for the fit to the power-law model and likelihood (LR) ratios with p-values are given for alternatives distributions.

Web of Science 1984 N = 437,225													
Time			Log-normal		Poisson		Exponential		Stretch Exp		Power law + cutoff		Support for power law
year	α	p	LR	p	LR	p	LR	p	LR	p	LR	p	
0	3.22	**0.50**	-0.16	0.88	8.71	**0.00**	8.59	**0.00**	1.20	0.62	-0.14	0.59	moderate
1	3.11	0.00	-2.04	**0.04**	9.37	**0.00**	8.08	**0.00**	-0.03	0.98	-5.28	**0.00**	with cut-off
2	3.17	0.08	-1.23	0.22	8.11	**0.00**	6.59	**0.00**	0.38	0.71	-1.98	**0.05**	with cut-off
3	3.07	0.00	-2.21	**0.03**	10.02	**0.00**	8.55	**0.00**	1.42	0.55	-6.00	**0.00**	with cut-off
4	3.12	0.03	-1.34	0.18	8.09	**0.00**	6.63	**0.00**	1.32	0.45	-1.91	**0.05**	with cut-off
5	3.09	0.00	-1.44	0.15	8.73	**0.00**	7.30	**0.00**	1.26	0.72	-2.31	**0.03**	with cut-off
6	3.06	0.00	-1.65	**0.10**	9.22	**0.00**	7.71	**0.00**	1.49	0.38	-2.93	**0.02**	with cut-off
7	3.03	0.00	-1.85	**0.06**	9.76	**0.00**	8.18	**0.00**	0.00	1.00	-3.71	**0.01**	with cut-off
8	3.04	0.00	-1.54	0.12	9.02	**0.00**	7.49	**0.00**	0.15	0.88	-2.36	**0.03**	with cut-off
9	3.23	**0.92**	0.25	0.80	5.47	**0.00**	4.63	**0.00**	1.48	0.14	0.00	1.00	good
10	3.22	**0.63**	0.44	0.66	5.37	**0.00**	4.65	**0.00**	1.56	0.12	0.00	1.00	good
11	3.19	**0.50**	0.11	0.91	5.43	**0.00**	4.70	**0.00**	1.46	0.15	0.00	1.00	good
12	3.22	**0.51**	0.50	0.62	5.30	**0.00**	4.77	**0.00**	1.78	**0.08**	0.00	1.00	good
13	3.00	0.00	-1.65	**0.10**	8.18	**0.00**	7.10	**0.00**	0.09	0.93	-2.34	**0.03**	with cut-off
14	2.98	0.00	-1.87	**0.06**	8.50	**0.00**	7.44	**0.00**	-0.13	0.90	-3.13	**0.01**	with cut-off
15	2.98	0.00	-1.79	**0.07**	8.31	**0.00**	7.26	**0.00**	-0.01	1.00	-2.82	**0.02**	with cut-off
16	2.98	0.00	-1.74	**0.08**	8.36	**0.00**	7.33	**0.00**	-0.12	0.84	-2.71	**0.02**	with cut-off
17	2.98	0.00	-1.75	**0.08**	8.14	**0.00**	7.00	**0.00**	-0.22	0.83	-2.64	**0.02**	with cut-off
18	3.17	**0.87**	0.52	0.60	5.39	**0.00**	4.88	**0.00**	1.79	**0.07**	0.00	1.00	good
19	3.16	**0.92**	0.32	0.75	5.70	**0.00**	5.12	**0.00**	1.71	**0.09**	0.00	1.00	good
20	3.15	**0.85**	0.23	0.82	5.88	**0.00**	5.25	**0.00**	1.77	**0.08**	0.00	1.00	good
21	3.14	**0.58**	0.24	0.81	5.96	**0.00**	5.29	**0.00**	1.84	**0.07**	0.00	1.00	good
22	3.13	**0.84**	0.20	0.84	6.11	**0.00**	5.42	**0.00**	1.73	**0.08**	0.00	1.00	good
23	3.11	**0.62**	0.11	0.92	6.23	**0.00**	5.49	**0.00**	1.64	0.10	0.00	0.97	good
24	3.10	**0.50**	0.16	0.87	6.31	**0.00**	5.59	**0.00**	1.71	**0.09**	0.00	0.98	good
25	3.08	**0.48**	-0.07	0.95	6.41	**0.00**	5.62	**0.00**	1.46	0.14	-0.01	0.88	moderate

The values in Table 3 summarize the results from Table 1. It gives the number of times that each type of support occurred in the evolution of the 1997 & 1998 citation distributions. Except for one instance the LR values for the Poisson, exponential and stretched exponential models were positive ruling them out as potential candidates. The LR values for the log-normal model were negative except for three instances and because they had insignificant p-values they could be ruled out as a possibility. In 98% of the instances the distributions had a moderate likelihood of being modeled a power law or a power law with an exponential cut-off. And the scaling exponents for the distributions decreased in magnitude with the passage of time from 3.36 and 3.21 to 3.06 and 2.99 in 1997 and 1998, respectively.

**Table 3:** Scopus—support for a power law distribution.

Support	1997	1998	Total	% Total
None	1	0	1	3%
Moderate	14	10	24	71%
Good	0	3	3	9%
Power law w/cut-off	3	3	6	18%

Table 4 summarizes the findings for five years of WoS data for which example data were given for 1984 in Table 2. In 93% of the instances the distributions had at least a moderate chance of being a power law or power law with exponential cut-off. And in 75% of cases of the WoS distributions were assigned a likelihood support of ‘good’ or power law with exponential cut-off.

**Table 4:** Web of Science—support for a power law distribution.

Support	1984	1985	1986	1987	1988	Total	% Total
None	0	3	0	5	0	8	7%
Moderate	2	4	5	6	5	22	18%
Good	11	6	0	9	12	38	32%
Power law w/cut-off	13	12	19	3	5	52	43%

Moreover as we move from observation times of 17 & 16 years using Scopus to 25 to 21 years using the WoS there is a greater chance the distributions will have a good fit to a power law or power law with exponential cut-off. These data support the notion that the distribution of the impact of knowledge has a reasonable likelihood of having scale-invariant properties.

Table 5 examines the field level distributions of citations to peer-reviewed papers indexed in the Scopus database in 1997 & 1998. Journals were assigned to one or more of 27 overlapping Scopus fields, one of 13 non-overlapping NSF fields and one of 13 non-overlapping MAPS fields. The data show that in 55–70% of the cases there is a ‘good’ likelihood that the field level citation distributions can be modeled by a power law or a power law with exponential cut-off.

**Table 5:** Support for power law distribution—field level analyses.

		Scopus		NSF		MAPS	
	Likelihood	No.	%	No.	%	No.	%
**1997**	none	39	8	5	2	11	5
	moderate	142	31	98	41	52	24
	good	25	5	10	4	21	10
	cut-off	253	55	123	53	137	62
**1998**	none	26	6	16	7	14	5
	moderate	172	40	85	38	64	31
	good	59	14	38	17	21	10
	cut-off	175	41	85	38	109	52
**Total**	none	65	7	21	5	25	5
	moderate	314	35	183	40	116	27
	good	84	9	48	10	42	10
	cut-off	428	48	210	45	246	57

These data illustrate that the impact of field level knowledge has a reasonable likelihood of having scale-invariant probability distributions too. Furthermore the result seems to be independent of whether papers are assigned to overlapping or non-overlapping fields

Scaling exponents with significant p-values for the field level distributions were examined at the end of the observation timeframe to determine how many fields had α < 3.0. Using the Scopus journal classification scheme 24 of the 54 (44%) field level distributions had α < 3.0. Also, 8 of the 24 (30%) fields had distributions with α < 3.0 both years. Using the NSF scheme 14 of 26 (54%) distributions had α < 3.0 and 4 of 13 (31%) fields had distributions with α < 3.0 both years. And using the MAPS scheme 10 of 28 (36%) of the distributions had α < 3.0 and 5 of 14 (36%) fields had distributions with α < 3.0 both years. Irrespective of the method used to assign papers to fields a significant number of distributions have α < 3.0 by the end of the observation time frame.

### 5.2 Impact-Size Scaling Correlations

**Fig 3:**
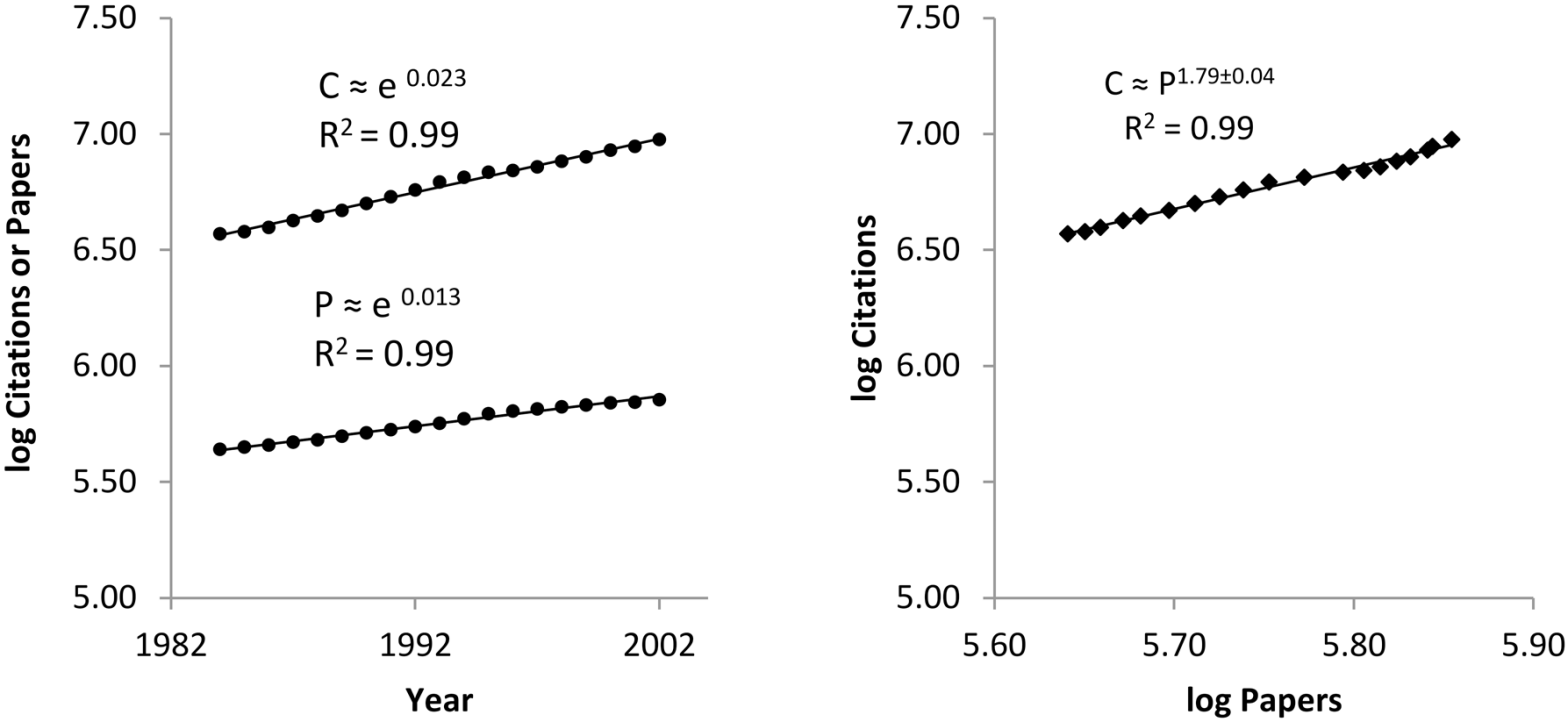
Scaling correlation between exponential growth of impact and size.

As shown earlier the simplest scaling correlation that any system will exhibit occurs between parameters that grow exponentially at the same time. Let’s examine how the increasing size of the global research system correlates with its impact. This is illustrated using WoS data because it covers a larger time frame. Fig 3a depicts the exponential growth over time on a log-linear scale of peer-reviewed papers and citations to these papers counted using a fixed 6 year time window. Fig 3b depicts the scaling correlation between citations and papers on a log-log scale.

The ratio of the exponential growth exponents, 0.023/0.013 = 1.77, is within the error limit range for the measured scaling exponent of 1.79±0.04. The scaling exponents tells us that on average for every doubling in peer-reviewed published output the impact was expected to increase by 2^1.79^ or 3.5 times. The scaling exponent, 1.79, is a scale-independent measure of the scale-invariant relative growth of the impact of the global research system over a 21 year time frame.

As discussed earlier scaling correlations have been found between impact and group size in the global research system at a point time. For example consider the scaling correlation between the impacts of research fields and their sizes based on Scopus data classified using the three journal classification schemes and done at two points in time. Table 6 gives the magnitudes of the scaling correlation across fields in 1997 and 1998 between number of citations and field sizes determined using the Scopus, NSF and MAPS journal classification methods.

**Table 6:** Scaling exponents for scaling correlation between impact and field sizes.

		1997		1998	
Scheme	No. Fields	α	R^2^	α	R^2^
Scopus	27	0.96 ± 0.09	0.83	0.96 ± 0.09	0.83
NSF	13	1.21 ± 0.08	0.91	1.19 ± 0.07	0.92
MAPS	13	1.27 ± 0.12	0.96	1.26 ± 0.11	0.96

The data show that the non-overlapping NSF and MAPS field assignments have α > 1.0 indicative of a self-organization. On the other hand the overlapping Scopus field assignment has α≈1.0 not indicative of self-organization.

Consider the following characteristics of the data. More than 50% of 1997–98 Scopus articles were assigned two or more Scopus general subject areas (fields). About 70% were assigned to two or more Scopus subject areas (subfields). Moreover, more than 18% were assigned to three or more fields and about 40% were assigned to 3 or more subfields. Assigning articles to 2 or more fields/subfields affects both the sizes of the fields/subfields and thier impact. Caution needs to be exercised as the methods used to classify groups in an innovation system can affect our view of its self-organizing structure.

## 6. Summary

The global research system is an example of an innovation system. Peer-reviewed publications and citations are used as measures of size and impact, respectively. The distribution of impact and the correlation between impact and size over time and at points in time were shown to have scale-invariant properties. Many outputs from innovation systems could have been used but as described earlier they have limitations. Citations and papers were used to illustrate scale-invariant concepts because the Web of Science and Scopus datasets are relatively clean and long time series are available. These measures have an extensive history of use in the study of innovation systems.

The global research system has the general characteristics of a complex system. Its adaptive nature distinguishes it from a complex physical system. At different levels of observation the evolution of the impact tended to be scale-invariant. The scaling exponent decreased toward a value of <3.0 as the distributions evolved. However, in some subfields the exponents became <3.0 within the first few years of their evolution.

Scale-invariant correlations exist between the growth of impact & size over time and between impact and size across fields & subfields at points in time. The scaling exponents of the later correlations are systemic measures of the ‘average impact’ of all the fields or subfields in the system. This scale-invariant correlation can be used as a reference function to calculate a scale-independent measure of how much impact a field or subfield is having relative to the average system impact.

A scale-invariant property has a unique characteristic. It is solely characterized by a power law f(x) = kx^α^ where α is a constant that quantifies the scale-invariant property. Due to its recursive or self-similar characteristics any natural community drawn from a population exhibiting a scale-invariant property will display that scale-invariant property too. Since the global research system is a complex innovation system with scale-invariant characteristics then any research system within it is likely to be a complex system with scale-invariant properties too.

This recursive property is useful. For example, it can be difficult to determine if the probability distribution of a property for smaller groups is scale-invariant. However, if it can clearly be shown that a property is scale-invariant at higher levels of aggregation then it can be said with increased certainty that it is likely scale-invariant at low levels too.

What are the implications for policy makers? Perhaps the most important take away is scale-invariance is natural and it is frequently found as an emergent property of a complex innovation system. Finding reliable measures to describe scale-invariant emergent properties is a concern. Many measures are based on population averages that are useful only when the scaling exponent of the underlying distribution is ≥ 3.0. Generally speaking real-world scale-invariant distributions tend to become <3.0 as the system evolves. Moreover, as group size decreases it is more likely to become <3.0 early in its evolution. On the other hand scale-independent measures based on emergent properties are useful throughout the lifetime of the system irrespective of the magnitude of the variance. Furthermore, only a small number of scale-invariant functions are needed to create a scale-independent evolutionary model of properties of a complex innovation system.

Some scientometricians claim that measures based on standard field-normalization procedures that use average field citations as normalization factors work well in practice. This may be true for scale-invariant distributions with scaling exponents >3.0. However, few, if any, rigorous comparisons have been made on the effect that field normalized and scale-adjusted measures have on the rankings of performance measures from distributions with scaling exponents <3.0 or with a mixture of scaling exponents <3.0 & >3.0. Further research on this matter is warranted to ensure that policy makers are getting an accurate evidence- based view of the innovation systems that are the targets of their policies.

The focus of this article was to explore the question “What is a complex innovation system?” The current research on scale-invariant properties of innovation systems and methodologies for determining their existence was reviewed. Finally, proven methods were applied to measures of impact and size to illustrate a variety of scale-invariant properties of the global research system. The global research system has characteristics of a complex adaptive system and it exhibits a variety of scale-invariant properties indicative of self-organizing processes. Most, if not all, innovation systems are complex systems. Scale-independent evidence based measures capture naturally occurring but rarely quantified emergent properties of a dynamically evolving complex innovation system. Scale-independent measures and models would be useful tools to include in any basket of measures used to inform innovation policy.
